# Public health system response to emerging infectious respiratory outbreaks in Iran

**DOI:** 10.1080/16549716.2025.2491199

**Published:** 2025-05-29

**Authors:** Zahra Afshar Hosseinabadi, Nasrin Shaarbafchizadeh, Mostafa Amini-Rarani

**Affiliations:** a Organizational Development and Administrative Transformation Department, Kerman University of Medical Sciences, Kerman, Iran; b Hospital Management Research Center, Health Management Research Institute, Iran University of Medical Sciences, Tehran, Iran; c Social Determinants of Health Research Center, Isfahan University of Medical Sciences, Isfahan, Iran

**Keywords:** Intervention, public health, Pandemic, realist study, qualitative

## Abstract

**Background:**

Emerging infectious respiratory diseases present significant challenges to public health systems worldwide. The recent COVID-19 pandemic has revealed critical weaknesses in Iran’s healthcare infrastructure, particularly regarding surveillance and testing capabilities. During the pandemic, Iran faced severe consequences, including a high death toll and overwhelming demands on its healthcare system. This situation highlights the urgent need for a stronger public health system in the country.

**Objective:**

This study identifies interventions implemented in Iran’s public health system during respiratory disease pandemics, their context, mechanisms and outcome.

**Methods:**

A qualitative realist study was conducted using semi-structured interviews with 21 public health experts across various sectors. Data were analyzed through content-directed analysis using the CIMO (Context-Intervention-Mechanism-Outcome) approach and the SPRP (Strategic Preparedness and Response Plan) framework. Data collection occurred from March to June 2024.

**Results:**

Analysis revealed that factors such as individual behaviors, social capital, institutional settings, and political pressures significantly influenced intervention outcomes. Key interventions included enhanced risk communication strategies and the establishment of specialized respiratory disease centers. However, bureaucratic inefficiencies and resource limitations hindered effective responses. Additionally, continued investment in local diagnostic production is essential for maintaining national laboratory and vaccination capabilities.

**Conclusions:**

The findings underscore the necessity for systemic reforms in Iran’s public health framework to enhance preparedness for future pandemics. The realist approach provided insights into the complexities of intervention effectiveness, emphasizing the importance of context in shaping health outcomes. Strengthening primary healthcare and fostering inter-sectoral collaboration are essential for building a more resilient public health system capable of addressing emerging respiratory diseases effectively.

## PAPER CONTEXT

- **Main finding**: The study has revealed some crucial insights regarding Iran’s public healthcare interventions during the pandemic of emerging respiratory diseases. Additionally, the research highlights the pivotal role played by context and mechanisms in shaping the effectiveness of these interventions.
- **Added knowledge**: It has been found that a comprehensive understanding of context and the functioning of these interventions in different contexts is essential for improving public healthcare interventions and neglecting these factors can undermine the effectiveness of health initiatives, particularly during health crises such as emerging respiratory diseases.
- **Global health impact for policy and action**: These findings are important for policymakers as they can assist in developing policies that can help strengthen the public health system during the pandemic of emerging respiratory diseases.

## Background

The challenges posed by infectious diseases have become increasingly complex today, particularly with the emergence and resurgence of infectious respiratory diseases [1]. Since the detection of the first human coronavirus in the 1960s [2], the 2019-nCoV has emerged as the seventh coronavirus, following SARS-CoV and MERS-CoV, both of which caused serious illnesses [3,4]. The COVID-19 pandemic has resulted in over 700 million cases and 7 million deaths globally between 2019 and 2024 [5]. In addition, it has revealed health system challenges such as a lack of preparedness for surveillance, insufficient capacity for testing [6], and a shortage of healthcare professionals to conduct large-scale case investigations [7]. During a pandemic, the importance of a robust public health system becomes increasingly obvious as it focuses on prevention, surveillance, and community-wide interventions [8]. For example, the COVID-19 pandemic prompted governments worldwide to implement non-pharmaceutical interventions (NPIs), such as lockdowns, to mitigate the spread of the virus [9]. Unlike hospitals and healthcare facilities, which primarily deal with individual cases, a strong public health system emphasizes early detection, timely dissemination of information, and the implementation of population-level measures to control the spread of infectious diseases [10,11].

Iran’s public health system, like many others, had faced challenges in past outbreaks like SARS [12,13] and was significantly impacted by the COVID-19 pandemic from its onset [14]. During the COVID-19 pandemic, Iran faced significant challenges in managing respiratory pathogens due to its highly urbanized population, strained healthcare infrastructure [15,16], and international sanctions that limited to medical supplies [17]. Iran’s daily COVID-19 death toll exceeded 700 deaths per day during the height of the Delta variant wave in 2021 [18] and the total number of cases in Iran was about 7,627,186, and the number of deaths was a total of 146,811 as of October 2024 [5]. The pandemic overwhelmed Iran’s health system, which was already under pressure from limited intensive care units (ICUs), shortages of ventilators, and personal protective equipment (PPE). The country’s densely populated areas acted as hotspots for virus transmission [19]. Iran’s proximity to countries with high transmission rates, coupled with difficulties in implementing and maintaining strict quarantine measures, further exacerbated the spread, leading to an increased burden of the epidemic and a higher number of casualties [20].

This overwhelming burden highlighted the need for better preparedness and more resilient public health systems, as discussed in the COVID-19 Strategic Preparedness and Response Plan (SPRP) [21], and highlights the critical need for a stronger, more responsive public health system to address future respiratory pathogen threats.

While several studies have investigated the challenges and interventions within Iran’s public health system, particularly in the context of managing the system during the pandemic, few have comprehensively analyzed its long-term resilience. For example, Ghanbari et al. evaluated Iran’s alignment with the World Health Organization’s strategic preparedness plan, noting the historical implications and the need for systemic reforms to enhance health resilience [14]. Gouya et al. provide an overview of actions taken by the Iranian Ministry of Health to maintain essential health services while adapting to the pandemic, emphasizing the importance of strengthening primary healthcare systems and leveraging existing health infrastructure [22]. Tabrizi et al. focus on the role of primary healthcare during the pandemic, illustrating how Iran’s extensive network of health centers and community health workers facilitated service delivery despite significant pressures [23]. Najafi et al. stated that the application of principles related to disaster and emergency management – such as managing interactions both within and outside organizations, disaster risk management, and data management – played a crucial role during the COVID-19 pandemic. They highlighted that enhancing societal resilience, which includes improving adaptation skills and maintaining health and social participation, was a primary concern expressed by senior managers during this time [24].

However, none of these studies have employed a realist evaluation to identify contextual factors, underlying mechanisms, and resulting outcomes. The realist approach is a methodological approach that seeks to understand not only what works in a given context but also why and how certain interventions produce specific outcomes in particular situations [25]. Therefore, the absence of a realist study in existing studies leads us to aim for a comprehensive understanding of the dynamics between interventions, contextual factors, and outcomes in Iran’s public health system during the pandemic of emerging respiratory infectious diseases pandemic.

## Methods

### Study setting and design

This study was conducted within Iran’s public health system, overseen by the Ministry of Health’s Health Deputy. Each University of Medical Sciences has a Health Deputy responsible for public health and primary healthcare services [26]. These deputies follow a consistent structure, serving the majority of the population. Each province has at least one University of Medical Sciences dedicated to its population [27].

### Methodological approach

**Figure 1.**
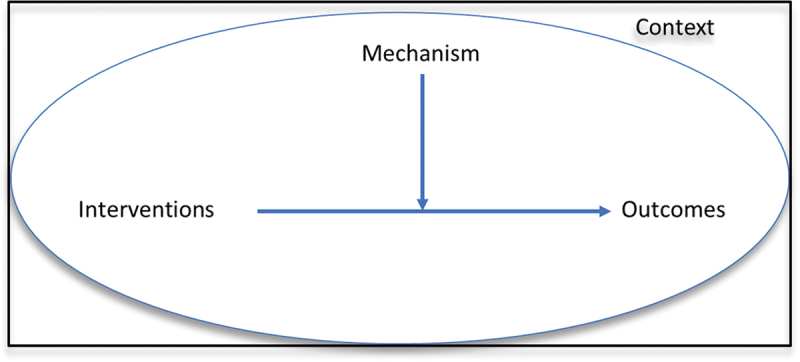
CIMO (context, intervention, mechanism, outcomes) configuration.

This study employs a realist methodology, a theory-driven strategy aimed at understanding causation and explaining the success or failure of health interventions [28]. It emphasizes the role of context in outcomes [29], using the CIMO (Context, Intervention, Mechanism, Outcome) framework to identify problems, introduce interventions, and understand outcomes [30,31]. (see Figure 1 for CIMO configuration).

Using the WHO’s ‘COVID-19 Strategic Preparedness and Response Plan’ to categorize interventions allows for a thorough evaluation of Iran’s health system. This approach helps identify both successes and areas needing improvement for future health emergencies [21,32]. Additionally, recognizing the importance of a more comprehensive, all-hazards strategy, the study aligns its findings with the Preparedness and Resilience for Emerging Threats (PRET) guideline, which highlights the necessity of system-wide readiness beyond just COVID-19 [33]

### Participation and data collection

Z.A.H., a PhD student at the time, conducted the interview with extensive qualitative research experience. Participants were informed of the study’s goals, and purposive sampling, including maximum variation and snowball techniques, was used to ensure a diverse range of health experts. We selected participants based on their expertise and role within the public health system, aiming to ensure a diversity of professional backgrounds and experiences rather than solely geographic diversity. We aimed for variation in their roles, institutions, and experiences rather than a strict geographical spread.

The inclusion criteria for this study required participants to have a minimum of three years of relevant experience within the public health system. The exclusion criteria included individuals who those lacking relevant experience.

Semi-structured interviews were conducted in-person (*n* = 21) or via phone (*n* = 2) between March and June 2024. Participants provided written informed consent, with phone interviewees signing and returning consent forms via email. Phone interviews focused on tone and pauses due to the lack of visual cues. Interviews lasted 45–90 minutes, averaging 60 minutes, and were audio-recorded and transcribed. Data collection continued until saturation was reached.

### Data analysis

Data was analyzed using directed content analysis and a realist approach to build CIMO configurations. Analysis began immediately after each interview. The first author summarized and coded data, with initial codes generated deductively from the Preparedness and Response Plan and the CIMO framework. Codes were reviewed, grouped into broader themes, and interpreted in relation to the research questions, forming the study’s findings. MAXQDA (version 18) software was used for data analysis, and the CIMOs were organized in Excel and discussed among the authors. The study’s quality was evaluated using four criteria by Lincoln and Guba: credibility, transferability, dependability, and confirmability [34]. To ensure credibility, the data gathering, coding, and analysis were extended over a span of approximately four months. An interview guide was developed to enhance the credibility of the interviews and the analysis of the transcripts. During the interviews, the interviewer took detailed notes, which were reviewed multiple times in conjunction with the coding process by the research team. Healthcare workers were purposefully selected based on their departments, and some of their quotations were incorporated into the study. A description of the study setting was provided to facilitate the transferability of the findings to other contexts, with data being collected and analyzed simultaneously. The study’s dependability was ensured through an auditing approach, wherein the authors collaborated with external auditors to provide complementary feedback. They cross-checked and examined inconsistencies, addressing them to reach a consensus. To enhance confirmability, the research team made a concerted effort to set aside their preconceptions, values, and theoretical biases as much as possible while interpreting the findings.

### Ethics and dissemination

Approval was obtained from the Ethics and Research Committee (ethics code: IR.MUI.NUREMA.REC.1401.021). Interviews were recorded with participants’ consent, and data was securely stored. Participant identities were anonymized, and study data will be stored securely for at least five years.

## Results

The study included 23 participants, comprising 16 males and 7 females, from various sectors within Iran’s public health system. These participants held diverse roles, including health deputies, faculty members, executive deputies, and experts in communicable diseases, environmental health, veterinary medicine, and disaster management. They were affiliated with organizations such as health centers, universities of medical sciences, and the Ministry of Health and Medical Education (MOHME), representing multiple cities, including Tehran, Isfahan, Kerman, Birjand, Tabriz, and Rafsanjan. The participants had extensive experience in public health, ranging from 10 to 35 years. Their backgrounds encompassed key areas essential for managing emerging respiratory disease outbreaks, such as health economics, policy implementation, disease prevention, veterinary medicine, and crisis response. By engaging with professionals at different levels of the health system, this study gathered insights that reflect both strategic and operational perspectives.

**Figure 2.**
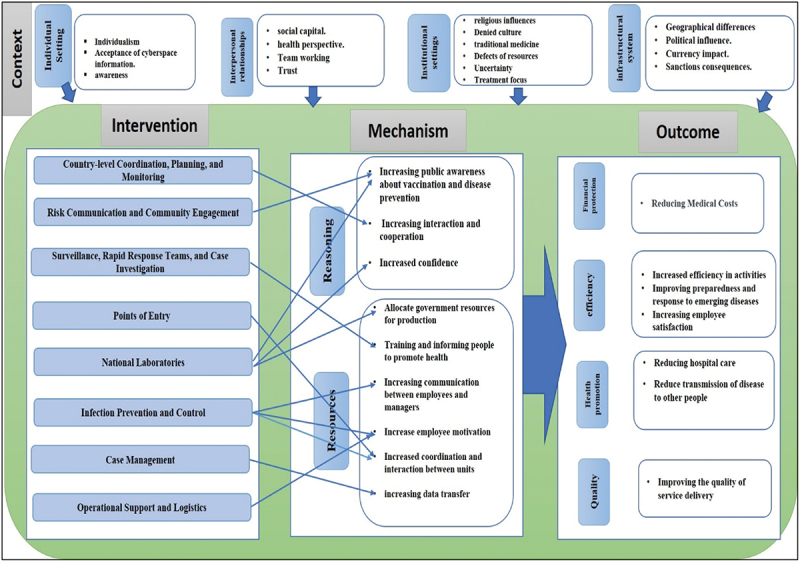
Configuration of CIMO framework based on the findings.

In Figure 2, the results of this study are presented and organized through the CIMO configuration, effectively illustrating the key components of the research findings.

## Context

Based on the definition of context in a realist study, the contextual factors that shape the implementation of the social programs include (1) the individual setting of capabilities of the key actors to take the intervention forward (e.g. values, roles, knowledge, purpose); (2) the interpersonal relationships supporting the intervention (e.g. communication, collaboration, network, influences); (3) the institutional settings (e.g. informal rules, organizational culture, leadership, resource allocation, local priorities); and (4) the external environment (e.g. political support). The context in which these interventions are conducted may limit or influence intervention decisions [35]. In this study, the contextual factors also have been grouped into four categories.

### Individual setting

In the individual setting of capabilities of the key actors, there exists a notable emphasis on individualism over social cohesion, leading to challenges in community-based health interventions. For example, one of the respondents believed:

Most people prioritize their personal interests and harbor skepticism towards communal benefits, particularly regarding the health system’s financial transactions. P2^1^^1^Participant Number 2.

The easy acceptance of any information in cyberspace poses both opportunities and risks, as misinformation can spread quickly, impacting public health behaviors. Additionally, a lack of awareness and attention to economic behaviors, coupled with psychological concerns and fear of societal repercussions, contributes to the barriers to implementing health interventions. The evolving needs and behavioral patterns of individuals further complicate public health strategies. According to one participant:

it is crucial to acknowledge that a lack of awareness in society, combined with psychological concerns and a fear of consequences, pose significant obstacles to the successful implementation of health interventions. P11

### Interpersonal relationships

Respondents noted reduced social capital and limited health perspective in policies, hindering collaborative efforts. The unclear role of passive defense and the absence of integrated health considerations impede holistic public health strategies. Lack of teamwork and decreased societal trust pose challenges to effective interventions. Participants mentioned that:

Our society faces cultural challenges as trust in virtual spaces and others’ words outweighs trust in our own. Falsehoods spread by authorities have led to a decline in social trust, which has affected our ability to convey messages effectively.P2

Many of the concepts at the macro level are infrastructural and metaphorical. For example, we don’t know how to ‘team up’ and there’s a rule of delusion in our subconscious. P11

### Institutional settings

Cultural factors and religious influences influence health behaviors and perceptions, as highlighted by interviewees. Government denial and reliance on traditional medicine and regional native cultures complicate evidence-based interventions. Barriers include resource distribution defects and uncertainty. Additionally, a treatment-focused approach and inadequate emphasis on primary healthcare hinder the development of a robust preventative public health system. As noted by one respondent:

SARS and influenza were controlled through prevention, and the same applies to COVID-19 and future respiratory diseases. Primary healthcare, accessible to all, bears the responsibility for prevention. Without a sound belief guiding our plans, our country risks crises. Thus, a belief-based strategy is vital to avert future crises. P12

### External environment

The COVID-19 pandemic presented numerous challenges to health system interventions, as reported by interviewees. These challenges were exacerbated by external factors, including geographical and demographic variations, currency fluctuations, political issues, and the impact of sanctions during epidemics. Additionally, political and security issues influenced decision-making and program implementation, impeding the effectiveness of public health interventions. In the words of participants:

Political factors and inflation impact the cost of raw materials for medicine and medical equipment. The dynamic political situation in our region, coupled with sanctions, can create additional constraints. P3

To implement contact tracing and restrictions effectively, ensuring favorable economic conditions and government support is crucial. If people can stay home and sustain themselves economically, disease transmission could be better managed, reducing spread. P1

## Interventions

In a realist approach, Interventions stem from a broader approach, encompassing strategies and implemented activities [36]. Health interventions refer to a broad set of coordinated strategies, policies, and actions implemented by health authorities to prevent, control, or mitigate the impact of public health threats. These interventions encompass both structural and operational measures aimed at improving pandemic response and ensuring public health resilience. In this study, health interventions were examined through the lens of the WHO’s COVID-19 Strategic Preparedness and Response Plan (SPRP) and Preparedness and Resilience for Emerging Threats (PRET) guideline, covering eight key dimensions.

### Country-level coordination, planning, and monitoring

The lack of multidisciplinary teams for implementing the necessary coordination to produce evidence in the field of IPC,^2^^2^Infection Prevention and Control. especially in developing countries, affects the rapid and successful response to the spread of infections and emerging diseases [37]. Iran did not explicitly conduct periodic Intra-Action Reviews (IARs) during the COVID-19 pandemic as described in global health guidelines. However, Iran established several strategies to respond to the pandemic, including the National Steering Committee and the National Committee for COVID-19 management, which facilitated inter-sectoral collaboration and decision-making [37]. Key recommendations from Iran’s response include strengthening institutional arrangements within the health system, improving coordination among policymakers, and enhancing communication channels across government sectors [37].

Moreover, the engagement of political leaders, senior officials, donors, non-governmental organizations, and universities in joint operational plans was another effective intervention. However, challenges like bureaucratic inefficiencies and lack of inter-sectoral collaboration hindered swift decision-making.

One participant highlighted: ‘The effective measures that were taken, as mentioned, were that they came and said the Ministry of Health should actually tell us from the command post position what it needs, how many resources it requires, and what it should do. The discussion was about what its treatment interventions would be, and this was good.’ P17

At first, there was a lack of equipment we needed and we didn’t have the resources to buy it. But over time، the Ministry of Health and the Partnership of various departments have come to help، like donors and industry have helped a lot. P5

### Risk communication and community engagement

The infodemic, the rapid spread of both accurate and inaccurate information during health crises like COVID-19, posed significant challenges globally, leading to confusion, mistrust in health authorities, and harmful behaviors [38]. Community engagement played a crucial role in addressing this issue [38]. In Iran traditional and social media platforms were used to disseminate accurate COVID-19 prevention information, while community-based initiatives mobilized local leaders and healthcare workers to promote reliable sources [39].

Respondents emphasized key interventions included raising awareness through diverse channels – radio, television, newspapers, healthcare personnel, and virtual platforms – alongside providing education at service points. Efforts also focused on improving diagnostic methods, tailoring care services to individual needs, and establishing specialized disease centerOne of respondents commented on this aspect, stating:

The centers in our structure were defined as the Selective Disease Center for Respiratory Diseases. not just for this disease, but for any disease in the form of an epidemic or pandemic. It is defined in our organizational structure. P19

In my idea public education efforts were the only intervention that proved to be effective in addressing the issues during pandemic. P9

### Surveillance, rapid response teams, and case Investigation

The One Health approach highlights the interconnectedness of human, animal, and environmental health, requiring comprehensive surveillance to detect and respond to emerging threats. This involves integrating laboratory diagnostics with epidemiological data across sectors to enhance public health strategies [40]. Advanced diagnostic techniques, such as wastewater testing, and strengthened national laboratory systems improve disease reporting and coordinated responses [39,40]

Iran ranks third in the Eastern Mediterranean region (EMRO) in implementing an electronic health system, which tracks key indicators like birth and death rates [41]. To improve surveillance, Iran established specialized respiratory disease centers, though initial inconsistencies in diagnostic methods affected efficiency. One participant noted:

We gathered unnecessary information initially, not foreseeing its usefulness. However, various systems and apps have proven beneficial, facilitating coordination among the Department of Health and hospitals. (P17)

Another participant emphasized the importance of early intervention:

In veterinary practice, we know that early intervention is crucial in controlling the spread of infectious diseases. The same principle applies to human health during pandemics. A timely and well-implemented quarantine can reduce the spread, not just of human diseases, but zoonotic diseases as well, which can spill over into animal populations. (P22)

### Points of entry

During the COVID-19 pandemic, Iran implemented a risk-based inspection strategy at Points of Entry (PoEs) to mitigate virus spread. Screening regulations were delayed for four months due to logistical preparations but were enforced by late March 2020, with environmental health measures in airports and terminals. The Iranian Ministry of Health coordinated these efforts through a national COVID-19 headquarters, developing context-specific protocols. Despite limited testing capacity and initial lapses in active case finding, PoE controls became critical as international travel resumed [42,43]

One participant emphasized: ‘People would go outside, get infected, and spread the virus, leading to an increase in mortality. The fact that our death rate was high wasn’t without reason. Part of it was tied to the sanctions, and part of it was linked to economic problems. Those who say sanctions have no effect are talking nonsense – they know it has an impact, and they know how harmful its effects are, yet they still make such statements. These factors caused us to face numerous challenges’ (P12).

### National laboratories

Iran’s national laboratory network, comprising 560 multi-sectoral laboratories, supports respiratory pathogen diagnosis, surveillance, and variant identification using multiple sequencing platforms [44]. Over 55 million samples have been tested, with approximately 2,200 sequenced and shared in international databases [44]. The WHO has donated advanced genomic sequencing equipment to enhance screening for genetic variants, including SARS-CoV-2 [45]. Genome sequencing plays a vital role in genomic surveillance, enabling the detection of new SARS-CoV-2 variants, monitoring viral evolution, and informing public health measures [46]. Before Iran’s first official COVID-19 case on 19 February 2020, early diagnosis was delayed due to the unknown characteristics of the virus and the lack of specific diagnostic kits [37,46]. To address this, Iran partnered with knowledge-based companies to develop domestic testing kits, reducing reliance on foreign imports despite international sanctions.

One participant noted: ‘With all the restrictions and sanctions we had, a number of kits came in, and then the Iranian knowledge-based companies made the effort to produce diagnostic kits.’ (P18)

### Infection prevention and control (IPC)

Ensuring the safety of staff during an pandemic is crucial for maintaining the capacity of a country’s health system [47]. Participants highlighted the challenges in managing the health workforce within the public health system during the pandemic. The interventions aimed to strengthen the health workforce in Iran encompassed various aspects, including human resource supply and management, financial and spiritual support, and training. In terms of human resources, interventions involved recruiting and organizing staff, including retired personnel and volunteers for vaccination and disease screening. The utilization of volunteers, military forces, and additional staff facilitated by the Ministry of Health contributed to the workforce. Furthermore, the engagement of health representatives through recruitment and training further enhanced the workforce. Financial and spiritual support interventions included the presence of officials alongside employees, payment for increased workforce size and hours, and the provision of letters of encouragement during the pandemic. Training interventions focused on augmenting personnel skills through enhanced training. One respondent commented on this aspect, stating:

Our initial human resources intervention was establishing a multipurpose university headquarters beneficial beyond the current crisis. Midwives, for instance, received training in various nursing courses, including ICU, tailored to the disease’s demands. Rapid training was prioritized during COVID-19, swiftly transitioning paramedics to caregivers. P4

Just as veterinarians understand the need for quarantine in controlling animal outbreaks, these same principles apply to human health crises. A quarantine may not be a perfect solution, but it’s a critical tool in preventing uncontrolled spread when a health system is overwhelmed. P23

### Case management

Ensuring high standards of care and therapeutic interventions in infectious disease management is crucial for reducing prevalence and mortality [48]. Iran’s referral system played a vital role, with designated ambulances safely transporting suspected COVID-19 cases to hospitals, allowing healthcare facilities to prioritize severe cases effectively [49,50]

Despite extensive health education campaigns promoting vaccination and mask use, misinformation and cultural attitudes posed challenges. Belief in traditional medicine and economic hardships also affected adherence to infection prevention measures. One participant highlighted vaccination efforts:

A very good campaign was launched to promote the use of multivalent vaccines. The media were extensively utilized, and some community leaders, including religious figures, were involved to encourage people. (P10)

### Operational support and logistics

A comprehensive pandemic response requires not only a strong healthcare system but also efficient logistics to ensure the availability of reagents, medical supplies, and resources across essential sectors. Strengthening supply chains for diagnostic tools, personal protective equipment (PPE), and medications is critical. Additionally, coordination across sectors such as transportation, manufacturing, and public services plays a vital role in maintaining essential operations and ensuring timely distribution of resources. A well-integrated approach enhances resilience and improves the overall effectiveness of pandemic preparedness and response.

Many non-profit NGOs and governmental organizations contributed donations, along with support from the World Health Organization and the World Bank. While the World Bank’s loan faced delays, it provided significant assistance. P1

## Mechanisms

In the scientific realist approach, Pawson and Tilley have proposed their own understanding of mechanisms. In fact, mechanisms explain how program outputs result from the choices (reasoning) made by stakeholders and their ability (resources) to translate these choices into action. According to Pawson and Tilley (2005), a mechanism is not merely a causality variable but rather a theory representing the logic that outlines the potential of human resources and reasoning. They emphasize that mechanisms, while typically concealed, are genuine, and although not directly observable, users underscore their reality [51]. Based on our interview findings, interventions in Country-level Coordination, Planning, and Monitoring resulted in enhanced coordination and interaction between units, as well as closer relationships across different levels of health referral. In Risk communication and community engagement, interventions focused on training and informing individuals to promote health awareness, leading to an overall improvement in awareness levels. Mechanisms supporting interventions in providing National Laboratories included government resource allocation for production, increased public awareness about vaccination and disease prevention, and a boost in confidence in domestic vaccines. In health information systems, interventions resulted in improved information processes and increased data transfer. For instance, some interviewees expressed the following insights:

One of the opportunities that came up within health education was that people’s awareness increased. People used to pay less attention to health issues. But after the spread of the Coronavirus, health issues of the Corona Virus were the first topic of conversation in almost every home, all over the television, and radio, Health centers had different organs that taught. P13

Cooperation and collaboration between all sectors emerged. The actions of the military, volunteer forces, and the private sector have all been effective, and it is important that for health only the Ministry of Health is not responsible and everyone should cooperate, it has been proven that intersectoral cooperation and coordination emerged. The actions of the military, volunteer forces, and the private sector have all been effective, and it is important that not only the Ministry of Health is not responsible for health and everyone should cooperate. P10

Moreover, based on the participant’s ideas the interventions in financing heightened cooperation in different sectors. In addition, regarding health workforce interventions, some mechanisms such as increased interaction, cooperation, employee motivation, and communication between employees and managers.

Motivation is a crucial factor in the quality of work performed by healthcare professionals. Even a simple task such as administering an injection can be impacted by a lack of motivation. It’s the dedication of nurses and doctors that makes a difference in patient outcomes. Effective communication between managers and employees in all areas of the health system, along with fair compensation, can greatly improve motivation, leading to better quality and quantity of work. P8

## Outcomes

Outcomes in realist approach refers to intended, unintended, or unforeseen outcomes of interventions, including aspects such as sustainability, quality, and integration of services at the macro level. Participants believed that interventions and mechanisms have collectively led to increased efficiency in activities, enhanced ability to manage health crises, and improved preparedness and response to emerging infectious respiratory diseases. The pandemic has also improved the ability to diagnose and manage outbreaks, as well as reducing the need to import sample tests from foreign countries. Employee satisfaction and an overall improvement in the quality of service delivery were also achieved as main outcomes of interventions and related mechanisms in the public health system during the pandemic. Another significant outcome of interventions in the public health system was the reduction of medical costs by using fewer health services, decreasing the amount of hospital care needed, developing coordinated services, lowering the transmission of diseases to others, and diminishing the burden of disease. One of the interviewees stated:

One of the results of using more services of comprehensive health centers and preventive services is reducing the number of hospitalizations and thus reducing hospital costs. P21

## Discussion

Using a realist approach, this qualitative study reveals a complex relationship between contextual factors that shape public health interventions in Iran, ultimately influencing the effectiveness and outcomes of health initiatives. In most studies during the pandemic, the significance of contextual factors and mechanisms was often underestimated, leading to their exclusion in various research endeavors. For example, in the study titled ‘The Role of Telehealth During the COVID-19 Outbreak: A Systematic Review Based on Current Evidence,’ while discussing the effectiveness of telemedicine, the review lacks a detailed exploration of disparities in access to technology and internet services among different populations, which can significantly impact telemedicine’s efficacy [52].

Our focus was on exploring contextual factors affecting interventions in Iran’s public health system and the mechanisms that influence their effectiveness. Our objective was not only to highlight the relevance of these factors but also to compare findings with other studies. Through this comparative lens, we aim to explain how these contextual factors influence the design and success of interventions and the mechanisms employed during pandemics.

The International Health Regulations (IHR) core capacities emphasize the importance of country-level coordination and planning in responding to public health emergencies. Iran’s interventions in this regard aimed to enhance collaboration and interaction, aligning with the IHR core capacity requirements. Ţigănaşu (2023) suggested that cultural factors, such as the level of individualism within a population, may influence the transmission of COVID-19. In societies with higher levels of individualism, people tend to take independent and uncoordinated actions, whereas, in collectivist societies, government-issued directives are more readily followed by the population at large [53]

The recognition of reduced social capital and the lack of a health perspective in policies underscores the importance of collaborative efforts. Some interventions addressed this issue by engaging political leaders and improving cooperation and communication. Alfano (2022) suggested that social capital significantly shapes individuals’ perceptions of both their social circles and strangers, thereby influencing pandemic spread and adherence to non-pharmaceutical interventions [54].

Based on respondents’ opinions, cultural beliefs and governmental denial emerged as major challenges for Iran’s public health interventions. Lee (2021) observed that public health guidelines on protective measures varied in effectiveness across international jurisdictions and between cultural groups. The success of these measures depends heavily on the norms and beliefs that drive an individual’s adherence to such guidelines [55].

Additionally, belief in traditional medicine remained widespread in many parts of Iran, posing challenges to evidence-based interventions. Therefore, health initiatives emphasizing technology and research development sought to promote evidence-based practices. Chali [56] similarly concluded that in Ethiopia, the use of traditional medicine, including seeds and leaves, was prevalent during the pandemic. The most common method of administration was oral consumption, and the majority of medicinal plants were sourced from home gardens. The widespread reliance on traditional medicine posed significant challenges to pandemic control efforts [56]

Moreover, external environmental challenges, such as geographical and demographic variations, were also significant factors. Health information interventions reflected a commitment to addressing these challenges by optimizing data management processes. This aligns with IHR’s core capacity requirements related to surveillance and health information systems. In France, research findings suggested that a region’s socioeconomic level was moderately associated with mortality rates during the pandemic [57]. Similarly, Žmitek et al. (2021) found that in Slovenia, vitamin D supplementation increased significantly in some regions due to mass media campaigns promoting supplementation during COVID-19 [58].

The recognition of political and security considerations in decision-making is crucial. Governance and leadership interventions aim to strengthen leadership and enhance crisis management capabilities. Clement (2021) explores the complexity of policy responses, emphasizing that strategies for emerging pandemics like COVID-19 are shaped by existing political systems and the goals pursued by governing authorities [59].

Moreover, Iran’s coordination efforts were hindered by bureaucratic inefficiencies and limited inter-sectoral collaboration. This challenge, particularly delayed decision-making, was also observed in other studies, which highlighted that fragmented governance structures in many developing countries hampered pandemic response efforts [60]. In contrast, nations like South Korea benefited from a more centralized and agile response system, which enabled rapid decision-making and a more effective pandemic response [61].

Public health campaigns in Iran significantly raised awareness, though misinformation in cyberspace remained a challenge. Comparative studies emphasize the harmful role of misinformation. For instance, Taiwan successfully countered misinformation through rapid, transparent communication strategies that increased public trust [18]. Iran’s electronic health system enhanced public health surveillance but faced challenges in ensuring consistency across early diagnostic methods, mirroring difficulties observed in other resource-limited settings. Rwanda’s early adoption of mobile technology for contact tracing exemplifies how strong digital infrastructure can bolster surveillance capacity – an area in which Iran continues to face challenges in meeting IHR standards [62].

Iran made significant progress in expanding its diagnostic capacity, particularly through collaborations with local knowledge-based companies. This strategy aligns with Cuba’s approach, where domestic innovation played a crucial role in mitigating resource shortages amid the pandemic. Similarly, a study in India demonstrated that government-private sector collaboration helped overcome resource limitations, resembling Iran’s efforts in vaccine production despite sanctions [63].

Iran did impose partial lockdowns and quarantine restrictions to combat COVID-19, including closing schools, malls, and shrines, and banning gatherings. However, these measures were often implemented late and inconsistently, with significant economic concerns due to US sanctions and low oil prices, which limited the government’s ability to support the population during a full lockdown. The lack of effective and comprehensive lockdowns, combined with delayed responses and information concealment, led to severe outbreaks and widespread criticism [64]. A broader perspective on non-pharmaceutical interventions (NPIs), such as lockdowns, is provided by studies in other countries. For instance, research in Kazakhstan and Kyrgyzstan demonstrated that NPIs can effectively reduce the growth rate of new COVID-19 cases, emphasizing the importance of timely and coordinated interventions [9]. This underscores the need for Iran and other countries to refine their strategies based on international experiences and adapt to evolving pandemic conditions.

The healthcare workforce in Iran faced immense strain during the pandemic. Although Iran recruited volunteers and military personnel, a major limitation was the lack of comprehensive training and psychological support for healthcare workers. Similar trends were observed globally. A review of international responses showed that many countries grappled with healthcare workforce shortages, compounded by inadequate mental health support [65]. This highlights the critical need for a prepared and supported healthcare workforce, a key IHR core capacity.

Sanctions and economic instability hampered Iran’s resource allocation efforts. The country relied on external donors and organizations like the WHO and the World Bank, highlighting the importance of international collaboration during health crises. A Global Health review emphasized the role of international aid in mitigating the impact of sanctions in resource-poor settings, drawing comparisons with other nations facing similar logistical challenges.

Improving health system performance is essential to ensuring high-quality healthcare services. Key performance mechanisms include enhancing cooperation, interaction, and public confidence in disease prevention, as well as increasing public awareness. Li (2020) emphasized that raising awareness about social distancing and cultural considerations was crucial in controlling the pandemic in India [66]. Furthermore, strengthening learning and information resources, increasing departmental coordination, and motivating employees are essential for health system resilience. Reddy (2020) highlighted the importance of effective communication in fostering therapeutic relationships with COVID-19 patients and addressing the mental health needs of vulnerable populations [67]

## Conclusion

This study contributes significantly to the After Action Review (AAR) of Iran’s COVID-19 response by documenting critical interventions, context challenges, and health system limitations. By evaluating these interventions, the study provides insights into how Iran’s public health system managed the pandemic and highlights gaps in coordination, resource allocation, and community protection.

In conclusion, the interventions implemented in Iran’s public health system during the pandemic, as revealed in this study, highlight both challenges and successes across the Strategic Preparedness and Response Plan. Contextual factors, such as individualism, cultural beliefs, and political instability, significantly influenced the effectiveness of these interventions. Mechanisms that fostered collaboration, public awareness, and improved data systems contributed to positive outcomes, such as enhanced crisis response, reduced medical costs, and improved workforce capacity. However, ongoing issues, particularly around financing and inter-sectoral collaboration, must be addressed to strengthen the system’s resilience against future health crises. This comprehensive approach underscores the importance of adapting public health strategies to contextual realities and ensuring sustainable, evidence-based approaches for long-term success of Iran’s healthcare infrastructure.
